# PorMe: A validated open-source image-based pore size and porosity measurement tool for 3D-printed structures

**DOI:** 10.1016/j.mex.2025.103611

**Published:** 2025-09-11

**Authors:** José I. Contreras Raggio, Miguel Pardo, Bernhard Weisse, Juan F. Vivanco, Ameet K. Aiyangar

**Affiliations:** a Facultad de Ingeniería y Ciencias, Universidad Adolfo Ibáñez, Viña del Mar, Padre Hurtado 750. 2520000, Chile; b Swiss Federal Laboratories for Materials Science and Technology (EMPA), Dübendorf, Überlandstrasse 129. 8600, Switzerland; c Faculty of Medicine, University of Bern, Murtenstrasse 11, 3008, Switzerland; d Department of Diagnostic, Interventional and Paediatric Radiology (DIPR), Inselspital, University Hospital, Bern, Freiburgstrasse 10, 3010, Switzerland

**Keywords:** Polycaprolactone (PCL), Bioglass, Composite bio-scaffolds, Direct ink writing, Additive manufacturing, Image analysis

## Abstract

The paper presents a non-destructive image analysis tool for characterizing in situ the inner micro-architectural features of 3D-printed scaffolds for tissue engineering applications. The study aims to provide a high throughput method for assessing geometrical porous and fiber-based properties such as fiber diameter, orientation, pore size, and porosity. Through this study, an open-source image-based software has been created that uses images acquired in each vertical plane during the printing process. The images are automatically added one above the other and thus a series of three-dimensional geometric properties of the printed structures are analyzed. The method was validated by using 3D-cilindrical scaffolds of 10 mm diameter and 17 mm height, with pore size around 400 µm and porosity range of 40–60 %. It was demonstrated a high porosity prediction and homogeneous inner architecture between our current method, Archimedes-derived porosity, and a high standard microCT-based 3D analysis. This work also details the steps of the porosity measurement (PorMe) algorithm, which involves image acquisition, segmentation, invalid pore determination, pore size calculation, and porosity determination in a high-throughput and non-destructive manner.

In situ, non-destructive image analysis tool to assess scaffold geometrical properties

Layer-wise assessment of pore size, fiber diameter and orientation as opposed to just outer (top and bottom) layers

Open-source tools used to develop the image analysis tool, to be made available for the research community

## Graphical abstract

**Graphical abstract: Visual Overview of the PorMe Algorithm Flowchart:** This diagram simplifies the algorithmic process, highlighting key stages including image preprocessing, comprehensive analysis, and measurement of elements such as pore size and strut dimensions. Additionally, illustrative examples of the generated results are presented for clarity.

**Figure:**
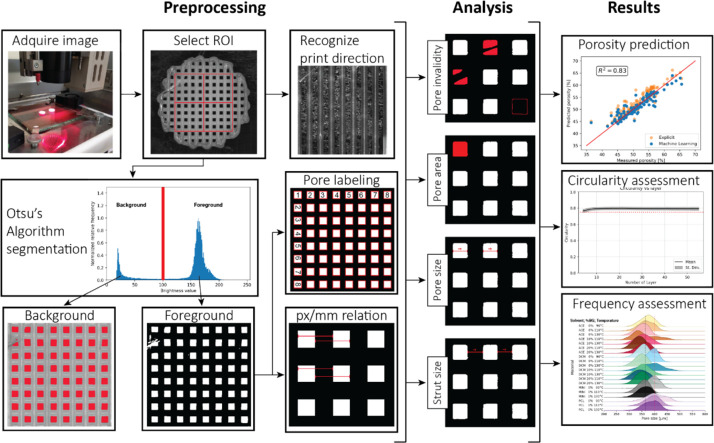

**Specifications table**

**Table:** 

**Subject area**	Materials Science
**More specific subject area**	3D Printing
**Name of your method**	PorMe
**Name and reference of original method**	*n/a*
**Resource availability**	*n/a*

## Background

Additive manufacturing has revolutionized scaffold production for various applications, including tissue engineering. The inner architecture of these scaffolds, encompassing properties like fiber diameter, orientation, pore size, and porosity, significantly influences their performance [1]. Achieving a reliable and efficient characterization method for these features is imperative [2,3].

Currently, the prevailing approach predominantly relies on manual measurements of the scaffold's external geometry [4,5]. Consequently, it suffers from inherent limitations, including low throughput and susceptibility to potential biases [6]. Furthermore, indirectly approximates the inner architecture based on the outer layers of the scaffold. When the inner layers are assessed, it involves slicing the fabricated specimens at multiple levels to ascertain layer-wise design printing fidelity. Thus, this traditional procedure is destructive, protracted, and labor-intensive [7,8]. While certain groups have made strides in implementing computer-assisted methodologies to streamline the process [6,9], it remains noteworthy that a comprehensive and explicit assessment of the inner structure can only be attained through destructive means.

To address these challenges, this paper introduces a non-destructive image analysis tool called "PorMe". This tool utilizes in-situ layer-by-layer images and open-source image-based software to provide high-throughput assessment of 3D-printed scaffolds micro-architectural properties.

## Method details

### Materials

The software was used to characterize the inner structure of the 3D printed structures of used in the study referenced here [10].

Commercially obtained polycaprolactone (PCL, Mn 45,000; Sigma-Aldrich) and bioactive glass (BG) were mixed following [10] using different compounding methods. PCL-BG inks were produced, analyzed and fabricated by Direct Ink Writing in a 3D-Bioplotter (EnvisionTEC, Germany) in form of cylindrical scaffolds of height 17 mm and diameter 10 mm.

The study evaluates the effect of ink preparation methods (4 levels: Acetone [Ace], Dichloromethane [DCM] and Mechanical compounding [Mini]), extrusion temperature (3 levels: 90, 110 and 130 °C) and BG content (3 levels: 0, 10 and 20 %). At least six specimens were 3D printed for each combination of parameters. These porous structures consisted of 54 continuous strut-style layers, with a pore size of 0.4 mm and porosity of 50 %. The code was used to analyze the over 300′000 pores in 216 fabricated specimens (4 × 3 × 3 × 6 = 216), enabling a comprehensive evaluation of the inner architecture.

### Image acquisition and PorMe analysis

Images acquired while printing were analyzed in order to characterize the inner quality of the printed structures in a non-destructive way.

PorMe is an image analyzer algorithm written in Python, which determines 3D-printed quality for orthogonal-shape scaffolds. Using acquired pictures while or after printing, the code measures a series of characteristics of the printed geometry such as strut thickness, spacing and pore size.

### Acquiring images

The camera attached to the bioplotter takes an image of each extruded layer once printed. The images are saved as PNG and stored in a folder with the name of the specimen and timestamp.

### Segmentation

**Fig. 1:**
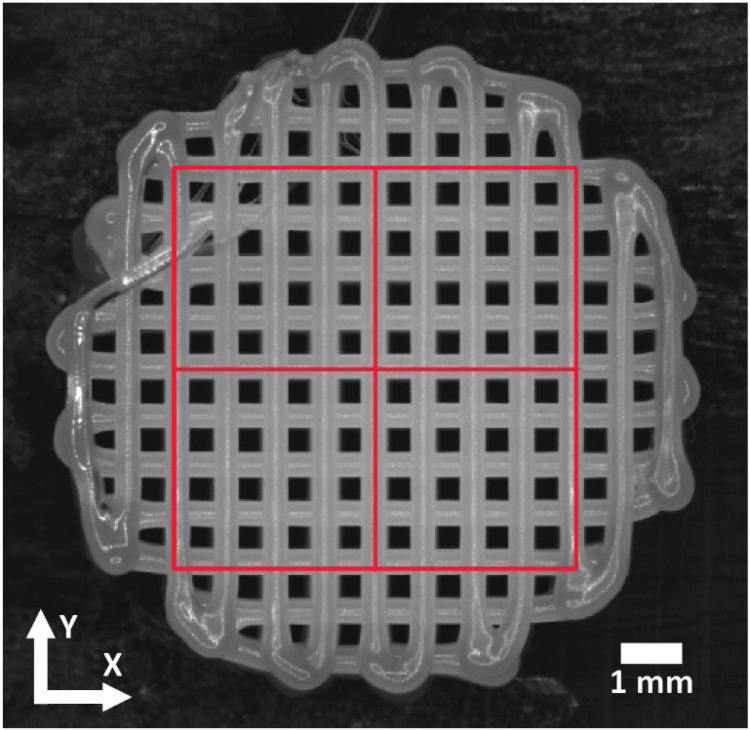
**Selected Region of Interest (ROI).** Red area represents the ROI of the inner part of the scaffold. Coordinates show printing direction and bar scale represents 1 mm.

**Fig. 2:**
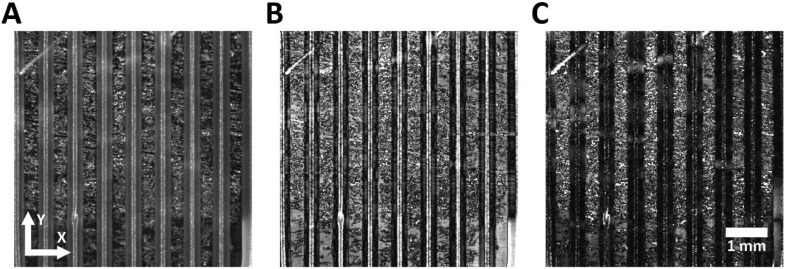
**Automatic printing direction detection.** Comparing of the original image (A) against its (B) X and (C) Y blurred difference, the darkest overall image corresponds to printing direction. Coordinates show printing direction and the scale bar represents 1 mm for all pictures.

The first step involves segmenting each image and identifying the inner-square portion of scaffolds. The folder of the images is selected, and a representative image is opened. The user then selects the region of interest (ROI) where the analysis will take place for the series of images (Fig. 1). The selection is cropped, and a second process automatically recognizes the printing direction in the first layer by comparing the layer against its X or Y blurred difference in the two orthogonal directions (Fig. 1) in the (horizontal) plane of the printed layer; the darkest overall image corresponds to printing direction (Fig. 2). The local brightness is corrected and normalized for the whole picture.

**Fig. 3:**
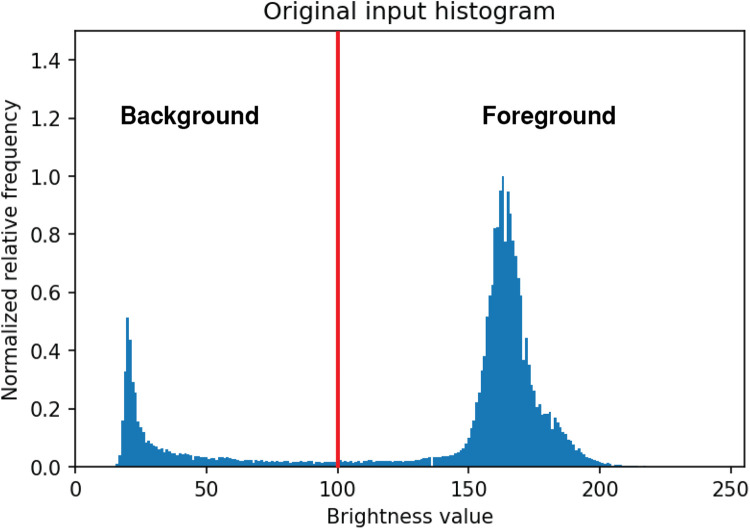
**Otsu´s algorithm applied on the image intensity histogram.** Where the red line determines the threshold value for the eliminated zone by Otsu´s thresholding algorithm, where values lower than the red line represent background and bigger values foreground.

The image is segmented into foreground and background by using different thresholding techniques. Images were segmented with Otsu´s algorithm method (Fig. 3). The method returns a single intensity threshold to separate foreground and background by minimizing the intra-class intensity variance [11].

The validity of the selected ROI coordinated is verify for potential user input errors. Subsequently, an analysis of the ROI borders is executed to ensure full pore incorporation, and if necessary, automatic adjustments are made to encompass the entire inner-square portion. Following this, a 2-D grid was performed on resulting images and all pores were unique labeled for future analysis.

### Invalid pores determination

**Fig. 4:**
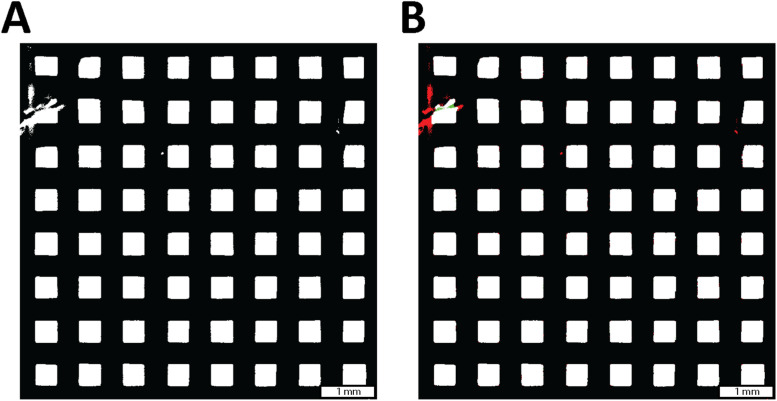
**Morphology process.** (A) Represents the unmodified image and (B) the detection of an error in a pore, where the green color is the material that is missing and the red color the artifact on the pore.

In order to effectively determine the pore size for each layer, a first step excluded invalid pores (Fig. 4). A pore is defined as the empty space between two adjacent printed struts. When this space deviates substantially from the designed pore geometry, an invalid pore is identified. Invalid pores represent an unintended pore geometry, characterized by extreme circularity values (>8 % deviation from of ideal circularity), small pore areas (area < 0.09 mm^2^) or the presence of multiples pores at a single location (split edges). The value is later exported as an integer or a percentage of the ratio between invalid and total pores for future analysis.

### Pore determination

The distance between pores is calculated and following the specifications of Bio-plotter (EnvisionTEC, Germany) manual, the pixel/mm relation is calculated.

**Fig. 5:**
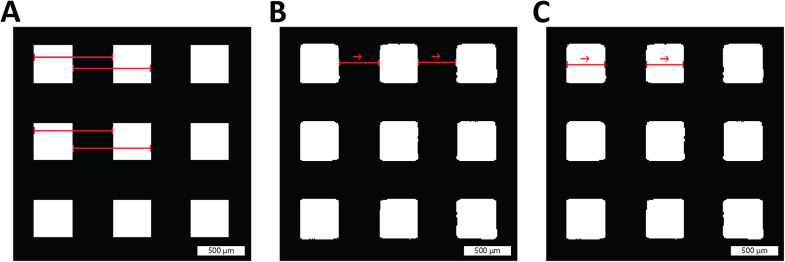
**Pore determination.** Where red lines show (A) the designed distance specify on the bioplotter configuration. (B) The pore distance, and (C) the pore size.

For each square pore, the area, perimeter, and length in both axes are calculated (Fig. 5) and the information is stored along with the position and identification of each pore. A subsequent process then calculates a series of characteristics such as circularity and pore distance (equivalent to apparent strut thickness). The information is later exported as a CSV for further analysis.

### Circularity measurement

Pore circularity was measured in order to quantitatively determine a value to represent the geometry of the pore. This term provides a measure for the sharpness of the orthogonal angle between two consecutive layers of struts Circularity is calculated following Eq. (1).

(1) $$Circularity=4\times \pi \times \frac{Pore\;area}{Pore\;perimeter{\;}^{2}}$$

Perfectly square pores have a circularity of ∼0.785, which is the ideal circularity value for the designed geometry. In contrast, a perfectly circular pore would exhibit a value of 1.

### Strut size determination

Furthermore, PorMe also outputs strut characteristics based on the 2D images. The apparent diameter of the strut is calculated as the distance between the pores, and total area is calculated as the non-pore area. These characteristics are calculated for each layer. The values are averaged and exported as a single value for each image (printed layer).

### Porosity analysis

**Fig. 6:**
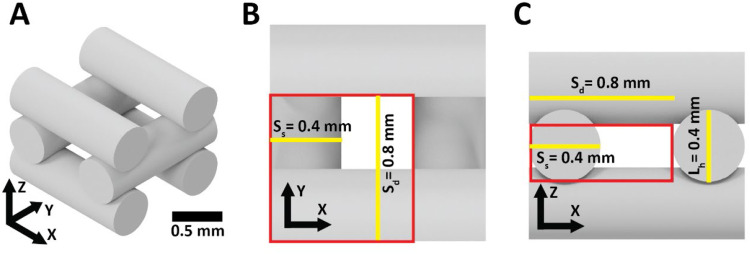
**Explicit formulation.** CAD representation of a pore (A) with (B) upper and (C) lateral view of it. ROI shows the zone for explicit calculation of local porosity by pore. Where: *S_s_* = strut size measured with the code from the images with an ideal size of 0.4 mm; *L_h_* = designed layer height; and *S_d_* = designed strut distance. Scale bar shows the dimensions in (A).

Local porosity of the region of interest of the sample can be mathematically approximated using Eq. (2) based on the strut size alone. Fig. 6 shows a segment of the ideal inner geometry of the scaffold, where the red area represents the region of interest for the porosity measurement. The pore size is used to represent the experimental inner structure, and Eq. (2) is used to calculate the porosity of the segment. The average of these local segments represents the porosity of the inner geometry of the scaffold.

(2) $$Porosity\;\left(\%\right)=100\times \left(1-\;({S_{s}}^{2}\times \pi \right)/\left(4\times L_{h}\times S_{d}\right))$$

where: *S_s_* = strut size measured with the code from the images; *L_h_* = designed layer height; and *S_d_* = designed strut distance configured on the Bio-plotter. For our configuration, *L_h_* was equivalent to 0.4 mm and *S_d_* to 0.8 mm (Fig. 6). With these values in Eq. (2), we can directly approximate porosity from the printed strut size as follows:

(3) $$Porosity\;\left(\%\right)=100-245.44\times {S_{s}}^{2}$$

Furthermore, using the acquired information, machine learning was used to predict the porosity. All acquired data were first preprocessed by filtering the parameters with a collinearity bigger than 0.8. Training data (70 %) with parameters – pore size, height and diameter – were used to train a Linear model with L1 penalty with cross validation (LassoCV with CV = 5 and an optimal alpha = 0.15). With a R^2^ = 0.81 for the training and R^2^ = 0.86 for the test split, the model coefficients for height and diameter were 0, meaning that the pore size is sufficient to predict the porosity. A new linear model was trained with an R^2^ of 0.86 (Eq. (4)).

(4) Porosity(%)=190.92×Poresize−17.94

## Method validation

### PorMe pore size analysis

**Fig. 7:**
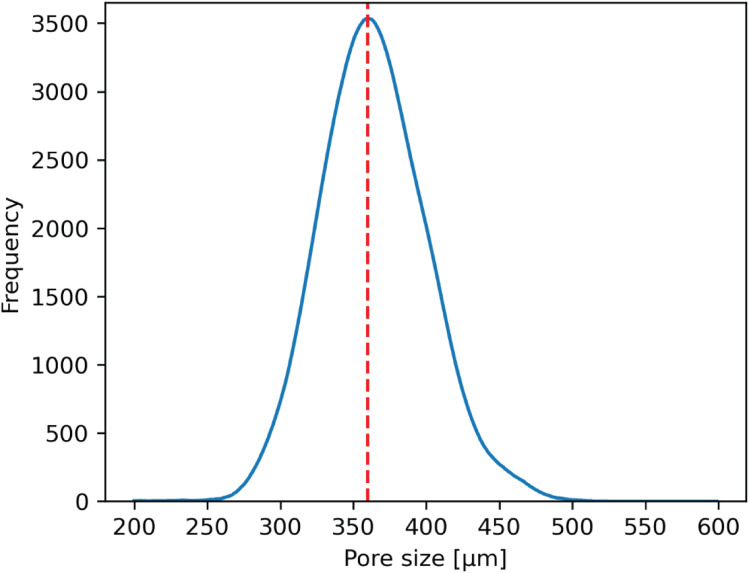
**Pore size histogram.** Frequency of pore size distribution for the >300′000 measure pores. Red dashed line represents the mode (= 360 µm) – the most frequently occurring pore size value within the displayed dataset.

**Fig. 8:**
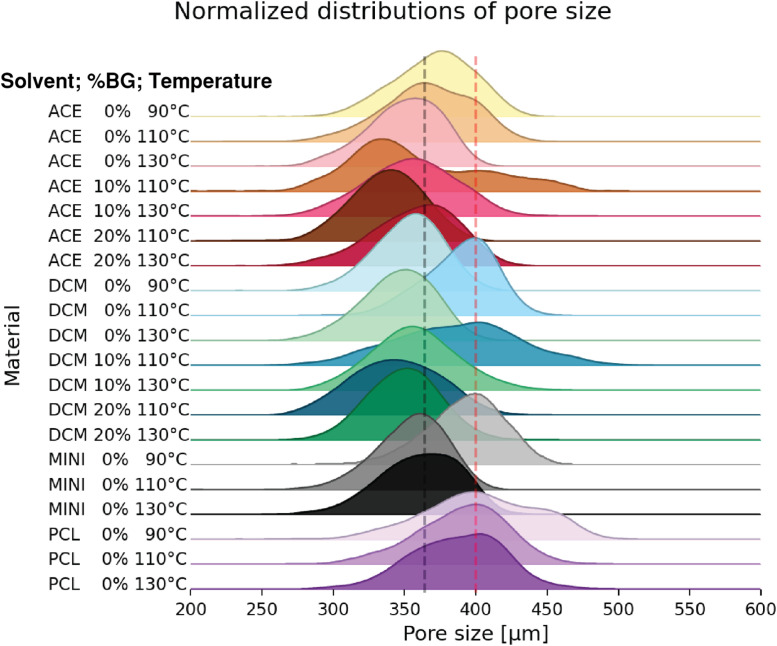
**Pore size histogram.** Frequency of pore size distribution for the different material combination. Height of the plots shows frequency percentage of the different pore sizes; red dashed line represents the designed pore size and grey dashed line the total average. Material labels follow: Ink preparation method, BG content; and extrusion temperature ( °C) respectively, where ink preparation methods are acetone (ACE), dichloromethane (DCM), mechanical compounding (MINI) and control of PCL (PCL).

The large amount of information allows a comprehensive analysis of the inner architecture. The over 300′000 pores analyzed can be represented in terms of frequency. Fig. 7 shows the frequency distribution of the pore size for the whole experiment, showing the homogenous printing process. Further analysis can be done by grouping the information by ink preparation method, BG content and extrusion temperature (Fig. 8), allowing to determine how process parameters affect the inner architecture distribution of the scaffold.

**Fig. 9:**
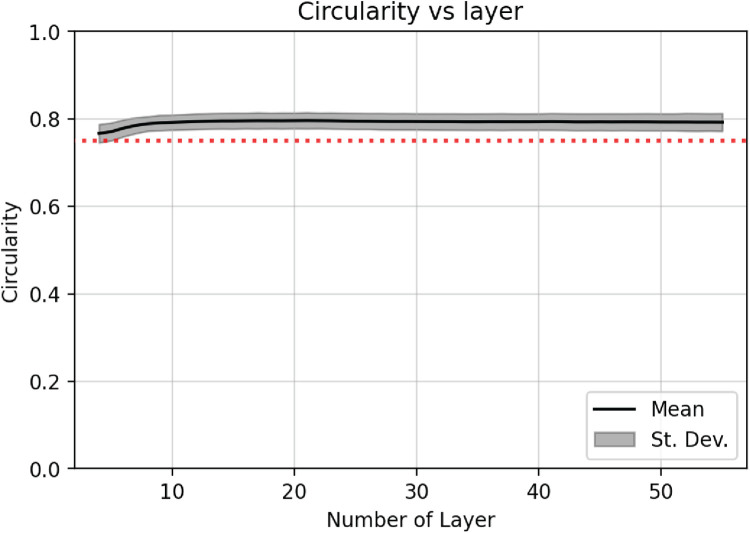
**Circularity changes along the printed layers.** Black line represents the cumulative mean for all measured pores and grey region represents the standard deviation**.** Red dotted line represents the ideal value for a square (∼0.785).

Furthermore, circularity analysis can be represented not only as a mean value for each sample as a whole, but also with respect to each printed layer. Fig. 9 shows how the circularity does not remain constant along the printing process and exhibits a high variation within the first 10 layers (Fig. 9-B). Previous work [12] conjectured that this variation may be attributed to changes in the cooling and solidification rates of successive printed layers of a large, finite-sized specimen.

### Inner pore size determination

PorMe analysis allows to effectively determine the inner pore size distribution. Furthermore, results can be compared with conventional technique, where just the upper and lower layer are analyzed and inner architecture is reported in terms of the average of these values. Therefore, when comparing both techniques, a big discrepancy can be seen in the inner distribution of the pore size.

Magnifying glass technique effectively acquire the pore size in both layers, having the first one usually a smaller value than the last. This difference can be attributed to the artifact produced by the printing bed attachment and thus the flattener of the strut and the reduction of the pore size. Thus, using the information of the first layer to predict the inner distribution can be misleading.

**Fig. 10:**
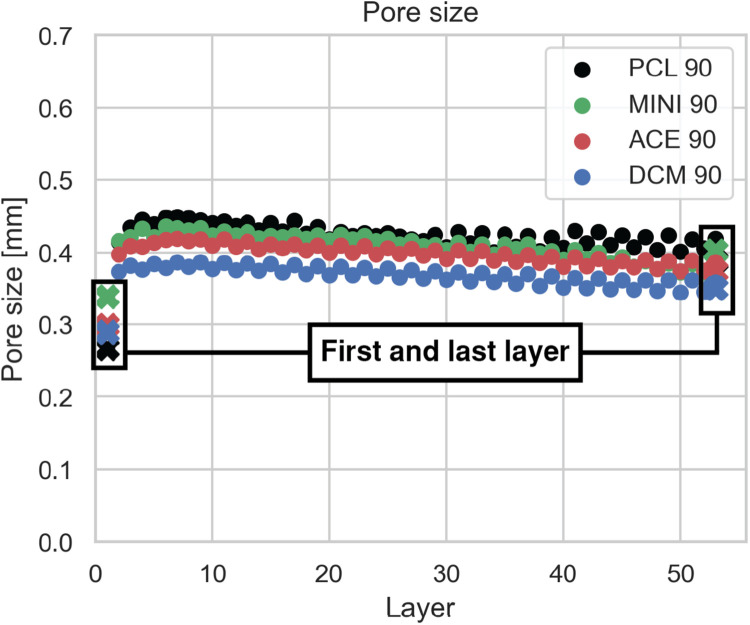
**PorMe performance.** Where (A) shows the difference between conventional technique and PorMe methodology. Crosses represent the data acquired with the conventional technique, wherein only pore size at the outer (first and last) layers are assessed. Labels show the ink preparation method and extrusion temperature ( °C) respectively.

Inner distribution cannot be confidentially extrapolated from first-and-last layer information. Fig. 10 shows the difference between the first and second layer, showing a big discrepancy between the values and a change in the inner trend. Conventional analysis would have reported an increasing trend on the pore size from ∼0.3 mm to ∼0.38 mm. Nevertheless, PorMe shows that from the second layer, the trend is actually slightly negative from a value of ∼0.4 mm to ∼0.38 mm. Moreover, a local increase can be found on the first 10 layers. Therefore, inner information is crucial for an accurate inner pore determination and the information cannot be extrapolated with just the outer layer information.

### Porosity prediction

Inner pore-size features were leveraged to predict bulk porosity. We first established an explicit analytic relation between mean inner pore size and porosity, then trained a sparsity-promoting regression model to identify the most informative image-derived predictors and estimate whole-sample porosity. Ground-truth porosity was measured on the fabricated specimens using the Archimedes method [1]. Open interconnectivity and the approximate homogeneity of the internal pore network were confirmed on a small subset via micro-CT reconstructions.

We trained a linear model with L1 regularization (Lasso) using k-fold cross-validation (*k* = 5). Prior to training, we removed highly collinear predictors (|ρ| > 0.8) and standardized features. Candidate inputs included image-derived pore size metrics, specimen height, and specimen diameter. Lasso selected pore size as the dominant predictor, yielding a sparse, interpretable model.

**Fig. 11:**
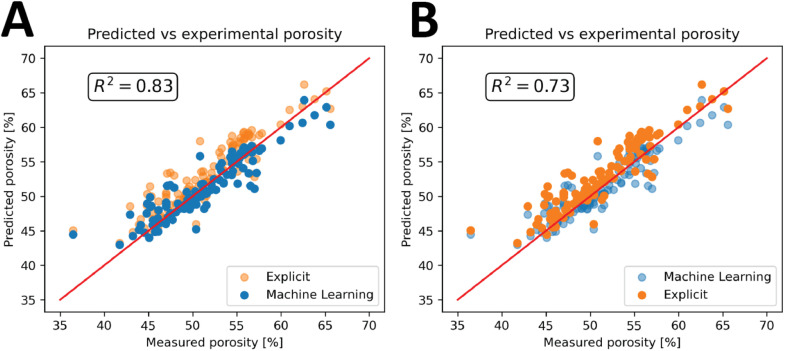
**Porosity prediction.** Where (A) highlights machine learning prediction (R^2^ = 0.83) and (B) the explicit approach (R^2^ = 0.73). The red line shows the main diagonal and R^2^ shows the coefficient of determination for the highlighted data.

The analysis determined that pore size can reasonably predict the porosity of the whole sample. Fig. 11 shows the difference between the explicit and the machine learning approach. Both approaches can effectively predict the porosity, with machine learning a better fit on the whole data set.

The PorMe algorithm is an effective tool, which allows the inner characterization of 3D printed scaffolds in a non-destructive manner. Furthermore, the ability to perform this assessment in situ, and in a high throughput manner allows for quick adjustment of the printing parameters, if necessary. As future work, we plan (i) a formal PorMe–micro-CT benchmark on a larger cohort and (ii) extending PorMe to operate directly on micro-CT z-stacks for cross-modal validation.

## Limitations

The software’s effectiveness relies on an orthogonal, square pore grid for accurately identifying and locating pores; non-grid architectures (e.g., rhombic, triangular, TPMS) may require further enhancements. The current version primarily analyzes the inner square ROI of scaffolds. In addition, PorMe assumes pore geometry along the build (z) direction (yz/xz planes) is approximately constant across layers; pronounced through-thickness deformation (e.g., sagging, interlayer necking, warping) may decouple z-direction changes from in-plane metrics and reduce accuracy.

## Conclusions

PorMe provides a non-destructive, in situ, and high-throughput route to quantify inner micro-architectural features (pore size, porosity, strut size, and circularity) of orthogonal 3D-printed scaffolds. Using layer-by-layer images, PorMe captures trends that are not accessible from outer-layer inspection alone and predicts porosity using an interpretable, sparsity-promoting linear model. Agreement with explicit-based analysis demonstrates that image-based characterization can offer an accurate, low-cost alternative for process monitoring and quality control. Current limitations include the assumption of orthogonal pore grids and a focus on the inner square ROI; future work will generalize detection to non-grid architectures and expand volumetric analyses

## Ethics statements

As our research does not involve human subjects, animal experiments, or data collected from social media platforms, we hereby declare that we do not require ethics approval for this study.

## Supplementary material and/or additional information

The PorMe source code and minimal example data are publicly available at https://github.com/JICRa/PorMe, released under the MIT License.

## CRediT authorship contribution statement

**José I. Contreras Raggio:** Conceptualization, Methodology, Software, Validation, Writing – original draft, Writing – review & editing, Visualization. **Miguel Pardo:** Methodology, Software, Validation. **Bernhard Weisse:** Writing – review & editing. **Juan F. Vivanco:** Writing – review & editing, Supervision. **Ameet K. Aiyangar:** Conceptualization, Writing – review & editing, Supervision, Funding acquisition.

## Declaration of competing interest

The authors declare that they have no known competing financial interests or personal relationships that could have appeared to influence the work reported in this paper.

## Data Availability

Data will be made available on request.
